# Tracking acute phase protein response during acute and chronic *Toxoplasma gondii* infection

**DOI:** 10.1186/s42826-019-0007-z

**Published:** 2019-07-24

**Authors:** Hasan Tarik Atmaca, Aycan Nuriye Gazyagci, Osman Safa Terzi, Gungor Cagdas Dincel, Tugce Sumer

**Affiliations:** 1 Department of Pathology, Balikesir University, Faculty of Veterinary Medicine, Balikesir, Turkey; 2 Department of Parasitology, Kirikkale University, Faculty of Veterinary Medicine, Kirikkale, Turkey; 3 Department of Internal Medicine, Ankara University, Faculty of Veterinary Medicine, Ankara, Turkey; 4 Eskil Vocational High School, University of Aksaray, Aksaray, Turkey; 5 Department of Pathology, Kirikkale University, Faculty of Veterinary Medicine, Kirikkale, Turkey

**Keywords:** *Toxoplasma gondii*, Encephalitis, Acute phase proteins, Acute, Chronic

## Abstract

Toxoplasmosis is a disease caused by the protozoan *Toxoplasma gondii*, which occurs worldwide in mammals and birds. Brain is the primary target organ because *Toxoplasma gondii* is a ubiquitous intracellular parasite that causes most frequently life-threatening encephalitis in immunocompromised patients. Relation of tissue cysts number, histopathology score and acute phase proteins were investigated. In this study, 36 mice are infected with Me49 strain of *Toxoplasma gondii.* The control group has 6 healthy mice. After inoculation of *Toxoplasma gondii*, at 10., 15., 20., 30., 45., 60. days, 6 each mice euthanized after collection of blood samples. Hemopexin, haptoglobulin, macroglobulin, serum amyloid A and clusterin levels are determined by ELISA. Then, brain tissues were investigated histopathologically and lesions were scored. The average cyst numbers were determined by counting three samples (25 μl each) of each brain homogenate under light microscopy. Inflammatory reaction was observed on day 10 days after inoculation (d.a.i.) The lesions were characterized by perivascular mononuclear cell infiltration, focal mononuclear cell infiltration in the meninges, and glial proliferation. Tissue cysts were observed in all *Toxoplasma gondii*-infected groups. The highest lesion score was observed at 60 d.a.i. And the most tissue cyst number were on day 30. d.a.i. Serum levels of hemopexin, haptoglobulin, macroglobulin, serum amyloid A and clusterin were significantly higher than the control group on day 10–20., 10., 10–30., 10.,10–45 d.a.i., respectively. High level of acute phase proteins in mice on certain days infected with *Toxoplasma gondii* was exhibited a relationship between brain lesions and tissue cysts.

## Introduction

*Toxoplasma gondii* is a parasitic disease caused by apicomplexan protozoan and disease named as Toxoplasmosis which occurs in mammals and birds [1]. Chronic *Toxoplasma gondii* infection primarily affects the brain and can cause fatal toxoplasmic encephalitis in immunocompromised patients [2]. Within the brain, *Toxoplasma gondii* invades astrocytes, neurons, and other neuroglia [3]. In mice, common histopathological findings are focal or diffuse inflammatory foci, blood-vessel cuffing and inflammatory cell infiltrates in the meninges and presence of parasite tissue cysts [4].

Tissue cysts formed by avirulent strains may remain in the tissue for years. Immunity can not overcome chronic *Toxoplasma gondii* infection. Throughout the life of the host, tissue cysts rupture in different times. Releases of bradyzoites after ruptures are destroyed by the immune system of the host. Inflammatory reactions may block the multiplication of *Toxoplasma gondii* but the formation of new tissue cysts may be generated. *Toxoplasma gondii* only can only be isolated from patients secretion, excretion or body tissue obtained from biopsies or by administration to test animals, or with the fluid or postmortem pathological examination [5].

The acute phase response is the initially systemic reaction of the organism against inflammation and tissue injury. It is generated by tissue repairing mechanisms and various defences to prevent invasion and damage and for restoring homeostasis [6].

Acute phase response usually induced by inflammation that may be localized near to organism. However, the systemic acute phase response includes endocrine, neurological and metabolic changes giving rise to leucocytosis, the development of fever and changes in hepatic gene expression [7].

Acute phase proteins can be used for prognosis of diseases and monitoring of disease treatment process, general health screening, and for diagnosis of disease. In the presence of pathological lesions, The Acute phase proteins are highly sensitive while having a low specificity for a specific disease. The Acute phase proteins are now recognized as having an important role to play in the diagnosis of disease in animals, but during an acute phase response, there are significant differences in their concentrations between animals in the pathophysiological changes [8].

To the best of our knowledge, there is no document describing the changes in serum concentrations of acute phase proteins and inflammatory score and tissue cysts number in experimental mouse Toxoplasmic encephalitis. The purpose of this study was to evaluate the dependent changes and correlation in the serum concentrations of haptoglobin, α-macroglobulin, hemopexin, clusterin and serum amyloid A with tissue cysts number and inflammatory score in brain of mice experimentally infected by *Toxoplasma gondii*.

## Materials and methods

### Infection model

For experimental infections, mice were inoculated intraperitoneally (IP) with 15 ME-49 cysts suspended in 0.25 mL sterile physiologic saline according to [4]. All experimental procedures and animal manipulations in the present study were approved by The Animal Care Committee.

### Experimental procedures and tissue processing

Swiss albino mice were infected IP with 15 *Toxoplasma gondii* cysts. Groups of six mice were anesthetized with pentobarbital by IP injection, blood was obtained for sera collection and euthanized by cervical dislocation on 10, 15, 20, 30, 45, 60 days after infection (d.a.i.). The brains were collected for histopathological analysis. After harvesting, the brains were fixed in 10% buffered formalin and processed for paraffin embedding and sectioning. Tissue sections (4–5 μm in thickness) were obtained from each mouse brain and were mounted onto slides for histological examination.

### Histopathology

Photomicrographs were taken using an Olympus BX51 microscope equipped with a DP25 camera (Japan). The total number of focal or diffuse inflammatory foci was counted in a sagittal section within the Central Nervous System (CNS) previously described [9]; blood-vessel cuffing and inflammatory cell infiltrates in the meninges were also analyzed. The inflammatory score was represented as arbitrary units: 0–2, mild; 2–4, moderate; 4–6, severe; and above 6, very severe. All analyses were performed at 40× magnification.

### Determination of the number of tissue cysts

Half of brain (sagittal) tissue taken at necropsy to determine the number of cyst was used. The brain tissue was homogenized with 2 ml distilled water. The average cyst numbers was determined by counting three samples (25 μl each) of each brain homogenate under light microscopy.

#### *Toxoplasma gondii* immunohistochemistry

All immunohistochemical tests were performed using a streptavidin–biotin kit (Thermo Fisher Scientific). Diaminobenzidine (DAB) chromogen was used for color labeling, and and Mayer’s hematoxylin was used for background staining. Normal mouse serum was used in negative control staining procedure. After deparaffinization step, endogenous peroxidase blocking process was done with 3% hydrogen peroxide in methyl alcohol for 9–10 min. Antigen retrieval was done using Tris-buffered saline (TBS, pH 6.0) in a pressure cooker for 30 min. Then, protein blocking step was carried out for 8 min. After then, the slides were treated with primary antibody (polyclonal *Toxoplasma gondii* antibody*,* previously used in [4]) for 1 h, after, 30 min secondary antibody step and 30 min streptavidin step was done. Slides were washed lightly twice with PBS for 5 min in every step. Sections were treated in a controlled under microscope with DAB for 10 min. Then slides were evaluated under light microscope after counterstained with Mayer’s hematoxylin.

### Measurement of acute phase proteins by ELISA

Hemopexin, haptoglobulin, α-macroglobulin, serum amyloid A and clusterin levels were determined by ELISA. All measurements were performed according to the protocol specified by the kit manufacturer.

### Statistical analysis

Statistical analysis was performed according to One-way analysis of variance and Tukey’s Multiple Comparison Test and for analysis of correlation the Spearman Rank correlation test was used. For all analyses, *p* < 0.05 was considered significant. (GraphPad Prism version 6.00 for Windows, GraphPad Software, La Jolla California USA, www.graphpad.com) The values represent means ± S.E.M, *p < 0.05*.

## Results

### Number of tissue cysts

**Fig. 1:**
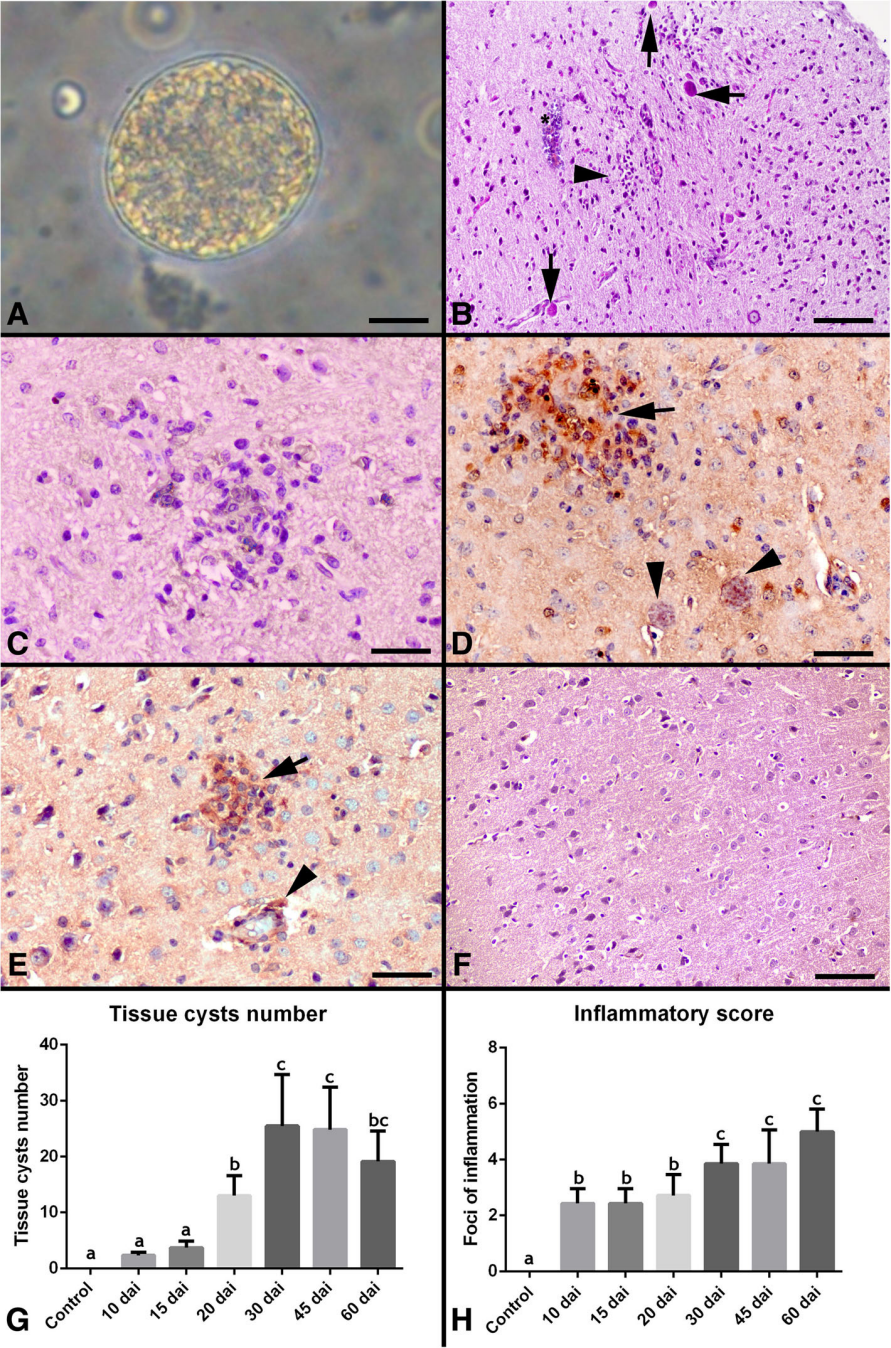
**a** *Toxoplasma gondii* tissue cyst. Scale bar = 20 μm. **b** Marked perivascular cuffing of mononuclear cells (asterisk), focal gliosis (arrow head), *Toxoplasma gondii* tissue cyst (arrows). Hematoxylin and eosin staining. Scale bar = 200 μm. **c**. Higher magnification of focal gliosis. Scale bar = 50 μm. **d**. Anti-*Toxoplasma gondii* immunopositivity in foci of gliosis (arrow), also tissue cyst was stained (arrow heads). IHC. DAB chromogen with hematoxylin counterstain. Scale bar = 100 μm. **e**. Anti-*Toxoplasma gondii* immunopositivity in foci of gliosis (arrows) and perivascular cell infiltration (arrow head). IHC. DAB chromogen with hematoxylin counterstain. Scale bar = 100 μm. **f**. Brain section from healthy animal. Hematoxylin and eosin staining. Scale bar = 200 μm. **g**. The number of tissue cysts number per brain (per 25 μl per brain homogenate) in 10, 15, 20, 30, 45, 60 day after infection. Values in a column suffixed with different letters are significantly different from each other. The values represent means ± S.E.M *P*<0.05 **h**. Comparison of total numbers of inflammatory foci. Values in a column suffixed with different letters are significantly different from each other. The values represent means ± S.E.M *P*<0.05

The average cyst numbers were determined by counting three samples (25 μl each) of each brain homogenate under light microscopy. *Toxoplasma gondii* tissue cyst (Fig. 1a) and the total number of tissue cysts observed are shown in Fig. 1g.

### Histopathology

The lesions were characterized by perivascular mononuclear cell infiltration, diffuse or focal mononuclear cell infiltration in the meninges and macrophages/microglia proliferation (Fig. 1b-c). The inflammatory scores are presented in Fig. 1h.

Tissue cysts were observed in all. *Toxoplasma gondii*-infected groups. Inflammatory lesions in the brain were more pronounced at the beginning of the infection and during established chronic infection.

### Immunohistochemistry (IHC)

Anti-*Toxoplasma gondii* immunopositivity were mainly observed in cells located in areas characterized by glial proliferation (Fig.1d-e) and perivascular mononuclear cell infiltration (Fig.1e) as demonstrated by histopathology. Additionally, the tissue cysts were shown immunolabeling (Fig. 1d).

### Serum acute phase proteins levels

**Fig. 2:**
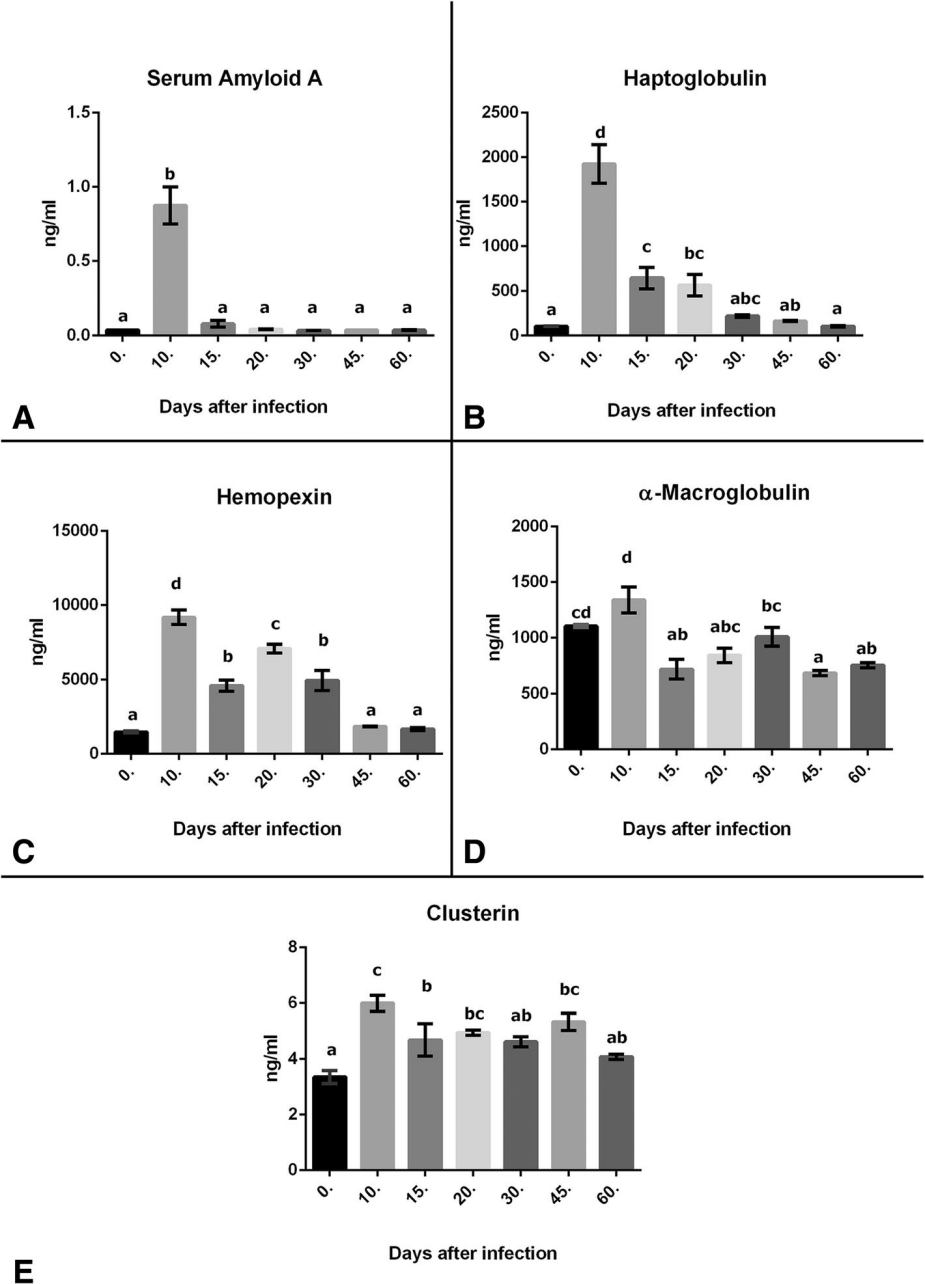
**a** Serum Amyloid A levels **b** Serum Haptoglobin levels **c** Serum Hemopexin levels **d** Serum α-Macroglobulin levels **e** Serum Clusterin levels. Statistical analysis was performed according to One-way analysis of variance and Tukey’s Multiple Comparison Test. Values in a column suffixed with different letters are significantly different from each other. The values represent means ± S.E.M. *P*<0.05

Serum levels of serum amyloid A, haptoglobulin, hemopexin, α-macroglobulin and clusterin were measured with ELISA method and results are shown in Fig. 2e, respectively.

Statistical analyses of each result are shown in related results in figures. After the correlation analysis of each acute proteins and tissue cysts number and inflammatory scores in days after infection times; it was observed that, at 10 d.a.i., positive correlations were found between serum concentration of haptoglobulin and serum amyloid A (r = 0.820, *p* = 0.045), and between Tissue cysts number and inflammatory score (r = 1, *p* = 0.001).

At 15 d.a.i., positive correlations were found between serum concentration of α-macroglobulin and serum amyloid A (r = 0.914, *p* = 0.011), α-macroglobulin and clusterin (r = 0.613, *p* = 0.179), haptoglobulin and hemopexin (r = 0.384, *p* = 0.452), haptoglobulin and clusterin (r = 0.708, *p* = 0.115), haptoglobulin and inflammatory score (r = 0.413, *p* = 0.415), serum amyloid A and clusterin (*r* = 0.469, *p* = 0.349). At 15 d.a.i., negative correlations were found between serum concentration of α-macroglobulin and hemopexin (r = − 0.250, *p* = 0.632), α-macroglobulin and inflammatory score (r = − 0.463, *p* = 0.355), serum amyloid A and hemopexin (r = − 0.286, *p* = 0.583), α-macroglobulin and tissue cysts number (r = − 0.386, *p* = 0.450), haptoglobulin and tissue cysts number (r = − 0.640, *p* = 0.171).

At 20 d.a.i., negative correlations were found between serum concentration of α-macroglobulin and haptoglobulin (r = − 0.943, *p* = 0.005), α-macroglobulin and hemopexin (r = − 0.891, *p* = 0.017), α-macroglobulin and clusterin (r = − 0.841, *p* = 0.036), haptoglobulin and infammatory score (r = − 0.820, *p* = 0.046), hemopexin and inflammatory score (r = − 0.933, *p* = 0.007). Positive correlations were found between serum concentration of haptoglobulin and hemopexin (r = 0.958, *p* = 0.003), haptoglobulin and clusterin (r = 0.873, *p* = 0.023), serum amyloid A and clusterin (r = 0.826, *p* = 0.043).

At 30 d.a.i., positive correlations were found between serum concentration of haptoglobulin and hemopexin (r = 0.902, *p* = 0.014), α-macroglobulin and serum amyloid A (r = 0.819, p = 0.046), haptoglobulin and inflammatory score (r = 0.834, *p* = 0.039). Negative correlations were found between serum concentration of hemopexin and tissue cysts number (r = − 0.818, *p* = 0.047), clusterin and inflammatory score (r = − 0.825, p = 0.043).

At 45 d.a.i., negative correlations were found between serum concentration of hemopexin and α-macroglobulin (*r* = − 0.985, *p* = 0.001), hemopexin and haptoglobulin (*r* = − 0.993, p = 0.001), hemopexin and clusterin (r = − 1, *p* = 0.001). Positive correlations were found between serum concentration of haptoglobulin and α-macroglobulin (r = 0.998, p = 0.001), clusterin and α-macroglobulin (r = 0.985, *p* = 0.001), clusterin and haptoglobulin (r = 0.993, *p* = 0.0001).

At 60 d.a.i., positive correlations were found between serum concentration of clusterin and haptoglobulin (r = 0.814, *p* = 0.048). Negative correlations were found between serum concentration of α-macroglobulin and inflammatory score (r = − 0.846, *p* = 0.034), haptoglobulin and inflammatory score (r = − 0.878, *p* = 0.021), clusterin and inflammatory score (r = − 0.909, *p* = 0.012).

## Discussion

Investigation of individual serum proteins in animals have been enhanced since over decades [10, 11]. This has largely been resulted by the notion that monitoring the levels of the acute phase proteins can serve as a useful tool to survey the innate immune responses to diseases [8].

Previous studies for significant changes in acute phase proteins in mice showed that Serum amyloid P and haptoglobin increased in experimental infection with Trypanosoma [12]. Serum amyloid P increased in experimental malaria infection [13]. Serum amyloid A and serum amyloid P increased with bacterial pneumonia [14]. In pigs, experimental or natural infection with *Actinobacillus pleuropneumoniae, Mycoplasma hyorhinis, Toxoplasma gondii, Bordetella bronchiseptica, Pasteurella multocida,* and porcine reproductive and respiratory syndrome virus leads to increased Haptoglobulin concentration in serum [11]. In this study, acute phase proteins importance in acute and chronic *Toxoplasma gondii* infection was investigated. It was thought that in the first 10 day period of post-infection serum concentrations of these proteins are different compared to other days and this difference can give an idea in diagnostic stage of the disease and in the determination of next steps of post-infection period. Likewise after *Toxoplasma gondii* infection, acute phase proteins assist determining the characters pathogen factors, that can cause secondary infections was observed.

In human medicine Acute phase proteins are used in the diagnosis of cardiovascular and autoimmune diseases, and organ transplantation in the management of cancer therapy and provide significant results [15, 16]. With the data obtained in this study for Toxoplasmosis investigation and treatment in the same way in the process is envisaged to be followed. Haptoglobulin and hemopexin is connected to heme in a strong way and help protect the tissues from active oxygen radicals and reduce oxidative stress through antioxidant effects [17, 18]. Also hemopexin blocks *Plasmodium falciparum* sporozoites.

In addition angiotensin II triggers localized inflammation and fibrosis with vasoconstrictor effect [19]. Hemopexin, which has ability to the drop feature regulation ATR-1R, protects vascular walls to vasoconstrictive effect caused from Angiotensin II. Haptoglobulin serum concentration, injury, burns, bacterial / parasitic infections or ischemic lesions 3–8 times increased between stands out with anti-inflammatory effect [20, 21]. Local and systemic activation of many cells through the release of prostaglandin synthesis by inducing the activation and migration of leukocytes, is involved in the regulation of cytokine models [21]. Another important task as maintaining haptoglobulin amyloidogenic proteins and abnormal structures of the dissolution of the proteins in the spaces between cells by preventing the accumulation of amyloid fibril formation [22]. On the other hand haptoglobulin binds to iron-binding protein and inhibits proliferation of intracellular pathogens with microbicidal mechanism [21, 23]. Haptoglobulin levels in chronic inflammatory condition are seen to be high. This is an important part in tissue repair haptoglobulin shows that in cases of systemic vasculitis compensation mechanism existing ischemia is involved in the development of collateral vessels [24]. In this study, hemopexin and haptoglobulin concentration in serum between the groups were found to be compatible with each other. In this case, the function shows a great similarity with each other for the fact that it is an expected result. The first 10 days post-infection of both a severe inflammatory process that begins to rise and parenchymal organs shows that severe tissue damage occurs. After infection 10th, 15th and 20th day groups, compared to control group of anti-inflammatory effects that stands out with haptoglobulin and hemopexin levels statistically higher (*p* < 0.05) even in these days,shows inflammatory process continued. After infection 30, 45 and 60 days towards normal levels of serum concentrations have decreased. Haptoglobulin and hemopexin levels after infection the first 10 day period after infection 45 and 60 days according to the statistical significant is too high for infection in the first 10 days are very important, and that in the process of disease pathogenesis research and tissue destruction in order to prevent a critical process shows that. Haptoglobulin and hemopexin in the 15th and 20th days after infection level, 60 days after infection by the statistically significant higher levels indicate that continued efforts be eliminated of intracellular pathogens. More than the maximum number of tissue cysts after infection 30, 45 and 60 days showed a decrease in the concentration of Haptoglobulin and hemopexin. This situation indicates that chronic phase develops. Hemopexin concentration (*p* < 0.05) were found to be more statistically from haptoglobulin. Consequently hemopexin appears to be more sensitive and responsive.

Major plasma globulin, α-macroglobulin, are involved as an plasma inhibitor in the fibrinolytic system and coagulation [25]. α-macroglobulin is expressed from hepatocytes and hepatic stellata cells [26]. Reducing collagenase activity in the liver, helps prevent fibrosis [27]. α-macroglobulin is involved for regulation of the immune system [28], in the process of cancer progression [29] and extracellular protein hemostasis [30]. Because of ability to bind peptides and particles foreign to the body provides a barrier against pathogens. Proteases that have exogenous and endogenous character, increase the virulence factors of pathogenicity of the parasites. α-macroglobulin has an additional feature like cleaning proteases of pathogenic microorganisms and parasites [31]. α-macroglobulin, can be used in prognosis and diagnosis stages of diseases due to the protection against infections [32]. Plasma concentrations of α-macroglobulin increases in many diseases such as pancreatitis and sepsis. In this study, a decrease was observed in α-macroglobulin concentration in the control group. The day 45 post-infection lowest level was determined. (*p* < 0,05). Significant differences were seen in α-macroglobulin concentrations groups 10 days and 15–45-60 days post infection. If inflammation scores are compared; in 45 and 60th days that, inflammation has the highest score, macroglobulin levels were found to be at their lowest level.

Clusterin was firstly isolated from rat testis with the in vitro studies [33]. Clusterin, that is a heterodimeric glycoprotein, and have roles in immune system regulation, cell adhesion, lipid transport, cell proliferation, regulation of apoptosis, tissue remodeling and interactions between cells [34, 35]. Also clusterin protein helps prevent physical and chemical stress by inhibiting the aggregation and precipitation. Clusterin also plays an important role in protecting of endoplasmic reticulum stress situations in cellular levels [36].

Clusterin has vital tasks such as, endothelial cell death triggered by light chain preventing the artery endothelial dysfunction, ensuring stabilization of the protein, and removal of the dissolved and damaged proteins [37]. In this study; significant differences, compared to control group, was determined in 10th and 45th days post-infection. It has been shown to reach the maximum serum concentration, 10 days after infection. 60 days after infection significantly lowest concentration is noted compared to day 10 post infection. Although the lowest concentration was seen in post infection 60 days, it is high compared to the healthy control group, but no significant expression was detected. In this case, the clusterin, that is positive acute phase proteins, has seen great significance expressed in the first 10 days of infection. In this case it is considered as a physical and chemical stress is formed during the acute phase of infections. If it is taken into consideration that endothelial cells are infected by microorganisms, cell death due to infection and possible function disorders also can be interpreted as the prevention of disturbances involved. As a result, severe increase in the acute phase for the regeneration of degradated cells, and in the chronic phase; continues above the normal level suggests that tissue healing process started. Clusterin, is an acute phase protein, which could agree that the stage of the infection and the process of interpretation.

Serum amyloid A, which is a pro-inflammatory cytokine for immune system cells, has been identified in mice [38]. Serum amyloid A contributes to immune system by triggering the adhesion, infiltration and migration of inflammatory cells by means of various receptors, such as G protein-coupled and formylpeptide receptors (FPRL1) [39]. Serum amyloid A is among the major acute phase proteins for dog and cattle [40]. Draws attention as a sensitive and specific marker for increased significantly during systemic infections in dogs. Serum amyloid A has a pro-inflammatory feature like cytokine. Triggers granulocyte colony stimulating factor (G-CSF) production in mouse macrophages, increases the release chemokines such as monocyte chemoattractant protein-1 (MCP-1) from monocytes and peripheral blood mononuclear cells [41, 42]. In addition, as the serum amyloid A response increases production of reactive oxygen species (ROS) from neutrophils [43]. The study findings were evaluated; compared to control group in the first 10 days Serum amyloid A concentrations raised very severe (*p* < 0.05). It’s levels has decreased in the same manner in 15th day after the infection almost equivalent to control group and continued to decline. Compared with inflammation scores, 10 and 15th days after infection, which has lowest inflammation score, serum amyloid A levels can be seen at maximum. This case shows us inflammatory cells called quickly to the region. Furthermore; these findings in mice for *Toxoplasma gondii* infection may indicate that the serum amyloid A is a major acute phase protein. It seems to be a sensitive marker against the severity of the inflammation occurring in the tissues. Also this case; maintain the continuity of the existing inflammation because of the inflammatory cells and taken thought to contribute to the chronicity process of inflammation. However, in very busy called inflammatory cells that express the cytokines considering the possibilities depending rose can also participate in the intensification of the inflammation.

Histopathological scoring revealed that inflammatory score increased from day 10 to day 60 d.a.i, as shown by an increase in the number tissue cysts from day 10 d.a.i to days 15, 20 and 30 d.a.i, which was associated with a time-dependent increase in inflammatory score expression. Dubey et al. [44] reported that *Toxoplasma gondii* tissue cysts formed in mice as early as 8 days after the inoculation with tachyzoites. And [4] confirmed it at 10 d.a.i. of *Toxoplasma gondii.* In this study we found the tissue cyst can be detected in 10 d.a.i. Therefore, the present study investigated brain lesion beginning on day 10 after infection. But positive correlations were only found between tissue cysts number and inflammatory score at 10 d.a.i. It can be done by cytes rupture. Because tissue cyst rupture increase the inflammatory score but decrease the number of tissue cyst in individual mouse. So it may lead that correlations were not found as positive. It was clearly showed that at 10 d.a.i the acute phase proteins increasing, especially α-Macroglobulin, serum amyloid A and haptoglobulin, at the beginning of tissue cysts formation and inflammation.

## Conclusion

In this study it is found that, serum amyloid A, haptoglobulin, hemopexin, α-macroglobulin and clusterin have been identified as major acute phase proteins and was found to quickly peaked in the first 10-day period after infection *Toxoplasma gondii* infection induced in mice. It is expected with this model has a major contribution in understanding the pathogenesis of the disease and diversification of patient approach in terms of natural infection. At the same time they give an idea of determining the severity of the disease and the period of infection. Present result confirm that hemopexin, haptoglobulin, α-macroglobulin, serum amyloid A and clusterin could be a potentially useful indicator in experimental *Toxoplasma gondii* infection studies or as a marker for disease character, because of correlation observed between its concentration in serum and tissue cysts number and inflammatory score.

## Data Availability

All relevant data are within this paper. Original data are available from the corresponding author (tarik.atmaca@balikesir.edu.tr) on reasonable request.

## Abbreviations

CNS: Central Nervous System
DAI: Days after infection
FPRL1: Formylpeptide receptors
G-CSF: Granulocyte Colony Stimulating Factor
IP: Intraperitoneally
MCP-1: Monocyte chemoattractant protein-1
ROS: Reactive oxygen species
SEM: Standard error of the mean
